# Supplementary material for: Are carbon emissions trading and green financial instruments synergistic? -Comprehensive quantitative research based on content analysis
Source: PLoS One. 2024 Mar 7;19(3):e0298601. doi: 10.1371/journal.pone.0298601 (PMC10919601; doi:10.1371/journal.pone.0298601)
Supplement: S1 Data — (ZIP) [file pone.0298601.s001.zip › Data/地级市Z_F工作报告.zip!/║■─╧/╓Ω╓▐╩╨/└┤╫╘╣1⁄2╓┌║┼í╛┬φ┐╦╩2╛▌═°í┐_2006.docx]

各位代表：

　　现在，我代表市人民政府向大会作工作报告，请予审议，并请市政协各位委员和其他列席同志提出意见。

　　一、2005年工作和“十五”计划完成情况

　　2005年，在市委的正确领导下，我们认真落实科学发展观，紧扣发展第一要务，突出经济工作三大重点，切实履行政府职责，全面实现了市十二届人大三次会议确定的工作目标。全年实现GDP520亿元，增长12.1%；完成财政总收入41.1亿元，增长21.9%，其中一般预算收入24.6亿元，增长21.2%；城镇居民人均可支配收入11230元，增长11.6%；农民人均纯收入3958元，增长11.9%。同时，10件大事进展顺利（详见附件1），10件实事全面完成（详见附件2）。

　　过去的一年，我们突出项目建设，投资拉动效应日益显现。全年共实施市以上重点项目45个，完成投资58.37亿元，其中23个基础设施项目完成41.82亿元，22个产业发展项目完成16.55亿元。株洲大道及栗雨工业园环线、金山大道、庐山路、嵩山路建成通车。纺织路等4条“市投市建”道路和南华路等19条“区投区建市补社助”道路顺利竣工。城市东、南、北三个“大门”拓展工程相继启动。红旗广场、响石广场及火车站广场等区域旧城改造顺利推进。龙泉污水处理厂投入运行。交通基础设施投资16亿元，106国道醴陵北段、攸县绕城线和炎陵县城至龙渣段提质改造全面完成。县城电网改造圆满结束。洮水水库建设进展顺利。株洲航电枢纽、醴潭高速正在加紧建设，长株、衡炎高速和网朱公路开工建设。同时，新上了智成化工16万吨纯碱、株化20万吨PVC、家润多商业广场、株洲（国储）物流中心等一批重点项目。全社会固定资产投资160亿元，增长23%，拉动GDP增长4.6个百分点。

　　过去的一年，我们推进开放调整，经济运行质量不断提升。市属国有企业改革进入尾声，一大批改革重组的工业和商贸企业重焕生机，全市规模以上工业实现增加值143亿元，增长15.8%，实现净赢利12亿元，增长20%。大力实施科技创新，全市新增高新技术企业26家，申请专利近800件，50个项目列入国家、省级科技开发计划；转化重大科技成果10项，2项获国家科技进步奖。全面铺开农村综合改革，落实各项支农惠农政策，全年农民人均增收423元。加快投资体制改革，促进了所有制结构调整，非公经济实现增加值229亿元，占GDP的比重提高到44%。积极调整商业网点布局，城乡市场更趋活跃，芦淞服饰市场进入全国十强，全市实现社会消费品零售总额182.1亿元，增长14.5%，物价涨幅控制在3.1%以内。不断加快开放步伐，提高了经济外向度。全年新批外资企业75家，实际利用外资1.6亿美元，增长25%，实际到位内资60亿元，增长33%；完成进出口总额6.6亿美元，其中出口5.43亿美元；“走出去”企业已有7家。全年共争取上级各类资金13.8亿元。开放调整步伐的加快，推动经济运行质量不断提升，财政总收入占GDP的比重、税收占财政总收入的比重分别提高到8%、74.5%。

　　过去的一年，我们加快农村“三化”，县域经济实力稳步增强。共投入6300多万元，大力推进了农业综合开发。实施水利建设“十百千万”工程，连续四次夺得省“芙蓉杯”。投入8300万元，退耕还林3.5万亩，保护国家级生态公益林260万亩，全市森林覆盖率57.5%。农业综合生产能力显著增强，全年粮食总产量达到186.4万吨，实现农业增加值70亿元，增长6%，经济作物产值占种植业产值比重、养殖业产值占农业总产值比重、非农收入占农民纯收入比重均超过50%。争取政府信用协议贷款额度30亿元，支持农业产业化项目。农产品加工企业发展到2600家，实现产值52亿元。投入2.7亿元，推进了以县城为重点的小城镇扩容提质，全市城镇化水平提高到42.5%。县域规模以上工业企业491家，实现增加值33.5亿元，增长25.1%，增幅高于城区10个百分点。五县（市）GDP、财政收入占全市的比重分别为51.9%、38.5%。

　　过去的一年，我们关注民生民利，社会和谐程度逐步提高。公共财政建设力度加大，财政支出进一步向教育、卫生、社会保障等公共领域倾斜。共投入2189万元，对9.5万名贫困学生实施“两免一补”（免杂费、免课本费、补助寄宿生生活费）；投入4856.3万元，推进了教育信息化；“普九”成果得到巩固，各类教育全面发展。市公共卫生紧急救援指挥中心和传染病防治大楼投入使用，农村药品“两网”建设全面实现“三年目标”，食品放心工程稳步推进。文化、体育、广电、档案、市志和其他事业蓬勃发展。就业、社会保障和扶贫救困工作得到加强。妇女儿童和慈善事业进步明显。人口与计划生育工作连续15年保持全省领先地位。社会治安形势平稳可控，安全生产形势稳中趋好。民兵预备役工作全面发展。市区环境质量明显改善，全年空气良好天数298天，增加101天，空气综合污染指数为2.867，下降了1.331，已经符合退出“全国十大空气污染城市”的条件；湘江水质基本达到国家Ⅲ类水质标准。土地市场与矿产资源开采管理秩序进一步规范。大力打击各类不法行为，依法维护了市场经济秩序。

　　过去的一年，我们坚持依法行政，政府自身建设得到加强。深入贯彻《行政许可法》，进一步提高了依法行政水平。认真执行市人大及其常委会的决议、决定。全年办理人大代表建议241件、政协委员提案251件，答复率、见面率均为100％，满意率99.5％。坚持以行政效能监察为中心，狠抓政务服务环境建设，出台了《关于进一步优化经济发展环境的十条意见》，同时大力查处了损害政务形象的案件405起。坚持民主科学决策，市政府领导牵头完成了32个课题的调研，全年出台规范性文件69个。严把公务员“录用关”，加大教育培训力度，狠抓反腐倡廉和纠风工作，提高了公务员队伍的整体素质。

　　2005年，是“十五”计划的最后一年。过去五年，在全市人民的共同努力下，“十五”计划确定的任务顺利完成。这五年，是我市经济持续健康发展，综合实力大幅提升的五年；是基础设施建设快速推进，城乡面貌变化最大的五年；是改革开放取得突破性进展，发展后劲显著增强的五年；是各项事业全面进步，人民群众得实惠最多的五年。

　　这五年，我们大力优化经济结构，发展水平明显提高。“十五”期间，全市GDP年均增长11.4%，高出计划目标0.4个百分点，2005年人均GDP14400元，超计划目标400元，位居全省第二。财政总收入和一般预算收入年均分别增长19.4％、18.2%，提前一年实现计划目标，五年翻一番。三次产业结构由17.3：46.7：36调整为13.3：49.6：37.1。工业化取得重大进展。2005年规模工业增加值是2000年的2.2倍，占GDP的比重五年提高了4.3个百分点，列全省第一。高新技术产业增加值占GDP的比重为13.2％，非农从业人员占全部从业人员的比重为57.5％，分别比2000年提高4.7、6.3个百分点。园区经济蓬勃发展。全市园区新上项目755个，2005年实现增加值占全市GDP的比重提高到19.8%。应该说，“十五”是我市发展史上最好的时期之一，为加快推进跨越发展，构建和谐社会奠定了坚实的基础。

　　这五年，我们加快基础设施建设，城乡面貌明显改观。累计完成全社会固定资产投资547.6亿元，是“九五”的2.4倍，年均增长22.4％。城市基础设施不断完善。建市以来最大的城市基础设施工程——总里程32.8公里、概算总投资23.7亿元的城市快速环道，已完成投资18.4亿元。投入70多亿元，新建和改造主次干道60条、广场10个、桥涵10座和一大批小街小巷，打造了21条样板街，新增道路面积约210万平方米。基本形成了湘江市区段4个防洪闭合圈，建成了3个防洪排渍站、2个污水处理厂、1个日供水能力达125万吨的自来水厂、1个可用16年的垃圾处理场、39座城市公厕、26个垃圾中转站。完成电信投资10多亿元，新增国际互联网宽带用户10万户，固定电话用户60万户。新增城市公交车429台，万人公交车拥有率提高到8.9标台。顺利通过了省级园林城市验收，人均公共绿地面积增加了2.8平方米。农村基础设施继续改善。共投入15亿元，完成各类水利工程18万处，治理病险水库485座，解决了9.3万人的饮水困难，治理水土流失面积495平方公里，改造中低产田99万亩，退耕还林51万亩，森林覆盖率提高了近4个百分点。广播电视“村村通”顺利推进。小城镇建设提速，共投入建设资金28亿元，全市城镇化水平提高了4.3个百分点。交通能源建设力度加大。累计完成交通基础设施投资35亿元，为“九五”的2.3倍。新建和改造公路1635公里，其中投入5.4亿元，完成了106国道株洲段全线284公里的提质改造；新增等级公路960公里，全市公路等级率由53%提高到66%，公路密度提高到每百平方公里36.6公里。高速公路新开工里程63.3公里。投资12亿多元，新建和改造变电站20多座，城市电缆入地180公里，农村电网改造正在加快推进。株洲航电枢纽三台机组并网发电。新建农村沼气池2万多个。通过五年的努力，全市城乡面貌焕然一新，为加快建设面积过百平方公里、人口过百万的特大城市，构筑了稳固的平台。

　　这五年，我们全面推进改革开放，发展活力明显增强。县属国有企业改革基本完成。市属国有企业改革成效显著，已完成改革的企业和已置换身份的职工，均超过计划任务的80%。在株中央、省属国有企业改革取得阶段性成果，已有8家企业剥离办社会职能，移交企业办学校18所、医院8所、离退休人员3.8万人。国有资产监管体系基本建立，行政机关经营性资产得到规范管理。农村税费改革取得实效，2005年已全部免征农业税；乡村消赤减债步伐加快，“两会一部”农民存款开始兑付，农村信用社改革初见成效。乡镇行政区划调整稳步推进，撤并乡镇26个、行政村344个。经过四轮行政审批制度改革，全市行政审批事项由1788项减少到377项。推行“五脱钩”，对中介机构的管理正在规范。财政改革力度加大，公共财政框架初步构建。按照“建管分离、条块结合、以块为主、条条监督”的思路，推进了城市管理体制改革。行业办改革取得圆满成功，经验在全省推广。投融资体制、粮食流通体制改革稳步推进。事业单位改革已经启动。要素市场逐步完善。金融运行整体平稳。同时，经济外向度不断提高，累计完成进出口总额25.5亿美元，年均增长12.2％；实际利用外资5.1亿美元，是“九五”的2.15倍，年均增长26.2％；引进内资170亿元，年均增长50％。同时，援藏和对口支援工作取得实效，分三批累计接收三峡移民1202人。改革开放的强力推进，为完善社会主义市场经济体制，推动株洲快步走向世界，拓展了广阔的空间。

　　这五年，我们协调发展各项事业，人民生活明显改善。共投入45.58亿元，促进了各级各类教育协调发展。“普九”成果进一步巩固。调减农村中小学633所，接收企业办学校33所，新批民办学校237所，教育布局逐步优化。职业院校发展到39所，其中高等职业技术学院5所。高等院校由5所发展到9所，湖南工业大学去“筹”全面成功，结束了我市没有多科性大学的历史。科技引领水平不断提高。全市共承担国家863计划项目19项（次），获省级以上科技成果105项，第5次跻身“全国科技进步先进城市”。新增中国工程院院士1名，企业博士后工作站3家，国家和省级工程技术研究中心（技术中心）5家。电动汽车示范运行课题顺利通过验收。株洲电力机车公司自主研发生产了“中华第一速”——中华之星电力机车。文化事业蓬勃发展，炎帝广场被评为“全国特色文化广场”，《沥沥太阳雨》获“全国五个一工程奖”。加大文明创建活动，2004年获“省文明城市”称号。公共卫生体系初步建立，累计投入7753万元，新建和改造了疾控中心6所、传染病防治区6个、乡镇卫生院24所，城市社区卫生覆盖面提高到90%。成功抵御了“非典”和高致病性禽流感的入侵。全民健身活动广泛开展，竞技体育水平不断提高，株洲运动健儿勇夺奥运奖牌2枚、全运会奖牌10枚、省运会金牌45.5枚。“四五”普法验收排名全省第一。第三次获得全国、全省社会治安综合治理先进地市称号，喜捧全国社会治安综合治理“长安杯”，央视《平安中国》专题推介了株洲经验。城镇居民人均可支配收入、农民人均纯收入分别增加4582元、1269元，年均实际增长11.1％、8％，城镇居民人均居住面积增加5.5平方米。全市累计归集住房公积金15.5亿元，发放6.56亿元。就业再就业规模扩大，新增城镇就业18万人，其中下岗失业人员再就业8.9万人。社会保障覆盖面拓宽，城镇职工基本养老、医疗、失业、工伤、生育保险全面实施，“两个确保”全面落实，共发放国有企业下岗职工基本生活保障金7.4亿元、离退休人员基本养老金32.1亿元、城镇低保金2.22亿元。救灾扶贫成效显著，累计投入救灾资金5342万元，扶贫开发资金7680万元，2.78万人解决温饱或越过贫困线。共投入资金近30亿元，连续两年分别实施并圆满完成了10件实事。同时，还获得了全国双拥模范城、人防工作先进城市、民族团结进步事业模范集体、无偿献血先进城市等国家级荣誉。各项事业的全面进步，为广大城乡居民共享发展成果，提高生活质量，提供了有力的保障。

　　各位代表，同志们，这些成绩的取得，是市委正确领导，市人大支持监督的结果，是市政协以及各民主党派、工商联、各人民团体参政议政和全市人民齐心协力，开拓进取的结果。在此，我代表市人民政府，向全体人大代表、政协委员、驻株部队全体官兵，向全市人民和所有关心、支持株洲发展的社会各界人士，表示衷心的感谢和崇高的敬意！

　　同时，我们也清醒地看到，我市经济社会发展中仍然存在不少困难和问题，主要表现为：产业发展缺乏大项目，新的经济增长点不多；体制、机制性障碍没有从根本上得到解决，市场主体活力不强；资金、能源等约束尚且存在，经济发展的瓶颈难以很快突破；少数部门和单位服务意识淡薄、办事效率不高；一些群众生活困难，弱势群体需要得到进一步关注；等等。这些问题，我们将采取有效措施，切实加以解决。

　　二、“十一五”发展基本构想

　　“十一五”既是全面建设小康社会承前启后的重要时期，也是推进跨越发展、构建和谐株洲的关键时期。发展机遇稍纵即逝，我们耽误不得；区域竞争日趋激烈，我们失误不起。为描绘好“十一五”发展蓝图，2004年5月，我们启动了《株洲市国民经济和社会发展第十一个五年规划纲要》（以下简称《规划纲要》）编制工作。去年6月，《规划纲要》初稿形成后，经过多次讨论和征求各方面意见，在与省“十一五”规划衔接的基础上，又提交市委九届十四次全会专题讨论和修改，形成了《规划纲要》草案，一并提请这次大会审议。

　　“十一五”时期，全市国民经济和社会发展的指导思想是：坚持以邓小平理论和“三个代表”重要思想为指导，全面贯彻落实科学发展观，加快“三化”进程，按照开放创新、扬长补短、以人为本、和谐发展的总体思路，突出推进跨越发展，构建和谐株洲的工作主题，实施产业兴市、科教先导、城镇带动、文化提升四大战略，努力建设开放、文明、繁荣、宜居的特大城市。

　　经济社会发展的主要预期目标是：

　　——GDP达到900亿元,年均增长12％；三次产业结构为9:52:39；非公经济增加值占GDP的比重达到60%；经济总量和人均GDP2007年在2000年基础上翻一番，2010年为2000年的三倍。

　　——财政总收入和一般预算收入年均增长13%，分别达到75亿元、46亿元。

　　——万元GDP能耗降低15%，能源和资源利用效率显著提高，城乡生态环境得到有效保护。

　　——城镇居民人均可支配收入年均增长10％，农村居民人均纯收入年均增长6％。

　　——城镇新增就业人数每年4万人以上，城镇登记失业率控制在５%以内；人口年自然增长率控制在7.5‰以内；城镇化率提高到51%。各项事业全面进步。

　　全面实现上述目标，我们将按照市委确定的总体思路和工作主题，不跑题，不走调，聚精会神搞建设，一心一意谋发展；不片面，不偏颇，统筹兼顾争多赢，包容共济促和谐。始终坚持以科学发展观统领经济社会发展全局,始终贯穿经济结构战略性调整这条主线，始终扣准改革开放这根主弦，始终把握提高人民群众生活水平这个主旨，全力实施“四大战略”，重点做好五个方面的工作。

　　（一）切实抓好“三农”工作，建设社会主义新农村

　　没有农民的小康，就没有全面的小康；没有农村的和谐，就不可能有全社会的和谐。建设社会主义新农村，我们将按照“生产发展、生活宽裕、乡风文明、村容整洁、管理民主”的总方针，全面加大支农惠农力度，努力使农民更加富裕，农业更大发展，农村更快变新。

　　完善基础设施。坚持城乡统筹协调发展，推动公用设施向农村延伸覆盖，加快农村交通、水利、通讯、电力、自来水等基础设施建设，改善农村生产生活条件。五年内，恢复和改善灌溉面积12万亩，新增有效灌溉面积6万亩，治理水土流失面积769平方公里；改造乡村公路5500公里，基本实现村村通水泥路或沥青路。

　　壮大农村产业。推进农业产业化经营，大力培育优势产业，加快发展潜力产业，扶持一批龙头企业。支持发展农村加工业、服务业等劳动密集型产业。改造提升农产品市场，鼓励发展农产品流通。到2010年，农业增加值达到86亿元，年均增长5%，农产品加工产值达到200亿元，农产品加工转化率提高到70%。

　　提高农民素质。加强职业教育和技能培训，培养有文化、懂技术、会经营的新型农民。支持农村劳动力转移，积极引导外出务工人员“爱乡回流”。培育各类专业合作经济组织，完善农村社会化服务体系，提高农民进入市场的组织化程度。

　　发展公共事业。大力改善农村中小学办学条件，到2007年全面实现“两免一补”。新建和改造一批乡镇卫生院，实施农村大病医疗救助制度，新型农村合作医疗覆盖面在80%以上。继续加强农村药品“两网”建设。提高农村“五保户”集中供养比例，符合条件的农村贫困居民全部纳入最低生活保障范围。

　　我们相信，经过5—10年的努力，农村生产力将得到明显解放，农村面貌将得到明显改观，农民生活将得到明显改善。到那时，广大农村居民与城市居民一样，户户用上清洁水，村村通上水泥路，看的是“有线”，联络有“程控”，出门有公交，就近有医疗，治病有保障，养老有依靠。

　　（二）加快推进新型工业化，提升产业发展水平

　　加快株洲发展，必须加快推进新型工业化进程，努力形成以高新技术产业为先导、先进制造业为支撑、现代服务业快速发展的产业格局。

　　突出集群发展，优化产业布局。重点发展交通装备制造、有色金属深加工、化工、陶瓷、农产品加工和服饰等六大产业集群，大力培育健康食品和生物医药、电子信息、新材料等高新技术产业，积极推动建材、花炮等传统产业优化升级，全市形成一区、三圈、一廊、九园的产业布局（株洲高新技术产业开发区；城市快速环道内的核心城区现代服务业经济圈、环道沿线区域的工业经济圈、环道外围的卫星城镇经济圈；106国道为轴线的县域经济走廊；欧洲工业园、清水塘循环经济工业区、金山民营科技园、建宁开发区、渌口开发区、醴陵陶瓷工业园、攸州工业区、云阳开发区和九龙工业区等九个工业园区）。同时，坚持“质量兴业、名牌强工”。到2010年，全市工业增加值达到408亿元，年均增长14%以上，六大产业集群加工销售收入达到1050亿元，形成一批有自主知识产权和知名品牌、主业突出、核心竞争力强的大企业、大集团，实现国有大中型企业与非公企业、县域工业与城区工业齐头并进。

　　突出科技创新，推动产业升级。健全有区域特色的科技创新体系，支持国家和省级工程技术研究中心（技术中心）、博士后工作站建设，力争在交通装备制造、新材料等优势产业的重点技术研发上取得新突破。重视科技成果转化，支持各类中试基地、示范基地建设，鼓励企业与高等院校、科研机构建立技术创新联合体。坚持以信息产业化带动产业信息化，鼓励企业运用高新技术、信息技术和先进适用技术改造传统产业，发展新兴产业，努力把株洲建成高新技术产业化程度高、区域综合竞争力强的创新型城市。力争到2010年，高新技术产业增加值占工业增加值的比重提高到40%，制造业增加值占工业增加值的比重提高到86%。

　　突出改造提升，发展现代服务业。服务业的加快发展，既是推进工业化的重要内容，也是工业现代化的重要标志。制定和完善促进服务业发展的政策措施，健全城乡市场体系，大力发展现代服务业，积极发展需求潜力大的产业，改造提升传统服务业。到2010年，全市社会消费品零售总额达到314亿元，年均增长13%，旅游总收入达到55亿元，占GDP的比重在6%以上，城镇居民人均居住面积增加到23平方米。

　　（三）合理定位城市功能，大力完善基础设施

　　区域经济社会的协调快速发展，离不开城市的带动，离不开基础设施的支撑。我们将大力实施城镇带动战略，在合理定位城市功能，增强城市带动力的同时，加快建设城乡一体的基础设施体系。

　　优化城镇布局。按照“东提西拓、合拢三角”的总体构想和第六次城市总体规划（修编），瞄准建设特大城市目标，中心城市基本形成“一江两岸双中心，二主五次七组团”的布局（依托湘江，在城市快速环道以内，重点建设河西新城中心和河东芦淞旧城中心，形成两个城市主组团，在城市快速环道沿线地区，重点建设栗雨、枫溪、荷塘、田心、石峰等五个城市次组团），巩固提升“两地一枢纽”地位（以高新技术产业为先导的新型工业基地、面向海内外华人的炎帝历史文化纪念地、南方重要交通枢纽）。同时，支持醴陵建成中等城市，攸县建成湘东南重要的经济次中心城市，加快建设其他3个县城和25个重点镇，形成功能配套、特色鲜明的城镇体系。

　　完善五大体系。1、综合交通体系。建设和改造“一专线、三干线、三站场”的铁路网络（武广客运专线；京广线、浙赣线、湘黔线；株洲站、株洲北站、株洲新站），建设“一纵五横三高速”的公路网络（S211株洲县南阳桥至衡东石岗公路；S313醴陵至株洲县伞铺段、S315攸县王家坊至大坪段、S320茶陵界首至界化垅段、S321炎陵县城至睦村段、网朱公路；醴潭、衡炎、长株高速）、“一江两水三港区”的水运体系（湘江；渌水、洣水；铜塘湾、白石港、凿石港）、以“两环两桥四门”为重点的市区交通网络（城市外环、内环；湘江四桥、五桥；东、南、西、北四个出入口）。2、市政公用设施体系。新建一批城市公厕和垃圾中转站，规划建设3个污水处理项目（河西、龙泉二期、枫溪），配套完善城市给排水管网，城市防洪标准提高到百年一遇。3、生态景观体系。建设“三带”（湘江生态景观带、快速环道城市绿化景观带、天元大道城市森林带），治理开发“四湖”（东湖、水竹湖、天鹅湖、云峰湖），改造“四港”（建宁港、白石港、霞湾港、枫溪港），完善“四园”（天台公园、凤凰公园、天池公园、荷塘公园），城市建成区绿化覆盖率提高到45%。4、工业园区设施体系。加大园区建设开发力度，提高服务质量，切实改变“重硬件轻软件、重规模轻配套”的现象。5、能源保障体系。重点加强主电网建设，提高天然气气化率，推广沼气等清洁能源。

　　促进可持续发展。控制人口增长，提高人口素质。严格落实国土政策，规范矿产资源开发秩序。加大环境污染治理力度，切实保护湘江“母亲河”，到2010年全市工业企业全面实现达标排放。鼓励清洁生产，大力发展循环经济，加快建设资源节约型、环境友好型社会。

　　（四）加快发展社会事业，促进经济社会全面进步

　　贯彻落实科学发展观，一个重要的方面，就是要让广大人民群众从经济社会协调发展中，得到更多实惠，实现更多需求。

　　优先发展教育事业。高度重视学前教育，2007年以前有条件的乡镇规划建设1所中心幼儿园，每个县（市）规划建设1所示范性幼儿园。巩固“普九”教育成果。进一步优化城乡教育布局，力争2008年基本普及高中阶段教育。支持湖南工业大学办成有特色、有声誉的多科性大学，支持中南林业科技大学株洲校区建设。充分发挥现有优势，加大扶持力度，把职业教育打造成为株洲教育的特色品牌。大力发展民办教育、特殊教育和社区教育。全市基本建成体系完整、布局合理、结构优化、发展均衡的国民教育体系和终身教育体系。

　　大力发展科技、人才事业。继续发挥政府投入的引导作用，鼓励企业多渠道筹措科技开发和产业化资金，形成全社会研发投入机制。到2010年，全市研究与开发支出占GDP的比重在2%以上。加强科学普及，提高全民科学素质。加大人才培养、引进和使用力度，建设好党政人才、经营管理人才、专业技术人才三支队伍。到2010年，全市人才总量达到32.4万人。

　　努力加强文化建设。构建市、区（县）、街道（乡镇）、社区（村）四级文化设施网络，支持建设株洲影剧院、东都商业文化广场等一批重点项目，新建和改造一批乡镇文化中心站。加快建设株洲广电中心，大力推进城区数字化电视整体转化和农村广播电视“户户通”。加强宽带通信网、数字电视网和下一代互联网建设，推进“三网融合”。继续开展“五下乡”活动，多出精品力作，丰富文化生活。壮大广电、文化、体育产业集团，构建出版物发行网络，形成文化产业支撑体系。规范发展文化娱乐业、体育中介业和体育彩票业。同时，大力培育具有本土特色的企业文化、社区文化，丰富文化内涵，提升文化品位；深入开展文明创建活动，增强发展的凝聚力和向心力。

　　不断提高医疗卫生水平。深化医疗体制改革，推进医疗资源重组和“医药分家”，加快市中心医院、各类特色医院及特色专科建设，建立以社区卫生服务中心和乡镇卫生院为基础，以综合医院、专科医院为支撑的全民医疗保健服务网络。到2010年，每千人拥有病床2.92张、执业医生1.95名，切实解决“看病难、看病贵”问题。努力控制重大传染病，积极防治职业病、地方病，加强妇幼卫生保健。

　　（五）不断改善公共服务，努力构建和谐社会

　　认真解决关系人民群众切身利益的就业、社会保障和安全等问题，实现居有其屋、劳有其岗、学有所用、困有所济、病有所医、老有所养，是构建和谐株洲的重要内容。

　　千方百计扩大就业。实施积极的就业政策，重视发展就业容量大的各类企业。鼓励自主创业和自谋职业，促进多种形式就业。完善就业服务体系，建立政府扶持、社会参与的职业技能培训机制。努力开拓国内外劳务市场，积极扩大劳务输出。加强劳动保障监察执法，规范用工行为。

　　着力完善社会保障体系。继续完善基本养老、医疗、失业、工伤、生育保险和住房公积金制度，提高社会保障能力。加大社会保险费的征缴力度，改进基本养老金计发办法，逐步做实个人账户。认真解决进城务工人员和失地农民的社会保障问题，逐步建立与经济发展水平相适应的农村初级社会保障体系。推进社会福利事业改革与发展，切实保障妇女、未成年人、老年人、残疾人的合法权益。规范和完善以最低生活保障为重点的社会救助制度，做好优抚安置工作，发展慈善事业。

　　全力维护社会稳定。加大社会治安综合治理力度，强化对食品、药品市场的监管，加强安全生产，建立健全灾情预警、防控和应急救援体系，提高应对突发事件的能力和水平。增强市民民主法制意识，营造健康文明的社会环境。加强和谐社区、和谐村镇建设，巩固社会和谐基础。落实民族宗教政策，正确处理新形势下的人民内部矛盾，畅通诉求渠道，完善社会利益协调和社会纠纷调处机制。加强现代国防和后备力量建设，巩固军民团结。

　　各位代表，同志们，“十一五”末，也就是到2010年，株洲的经济总量将近千亿，城市人口和面积“双过百”，并将建成国家园林城市、国家卫生城市、国家环保模范城市和中国优秀旅游城市。届时，一个综合实力更强、发展活力更足、城乡环境更美、人民生活更加富裕的株洲，必将呈现在我们的面前！

　　三、努力实现“十一五”良好开局

　　2006年，全市经济社会发展的主要预期目标是：实现GDP580亿元，增长12%，其中一、二、三产业分别增长5%、13.5%、12%；财政总收入和一般预算收入均增长13%；城镇居民人均可支配收入增长10%，农民人均纯收入增长6%；城镇登记失业率控制在4.5%以内；人口自然增长率控制在6.5‰以内；城镇化水平提高到44%；各项事业都有新的进步。实现“十一五”良好开局，我们将突出以下四个工作重点。

　　（一）坚持工业主导，加快“三化”进程

　　突出园区建设，大力推进工业化。重点发展先进制造业。支持企业加大科技创新力度，推动产业结构优化升级。支持太子奶生物栗雨工业园生产基地、中材集团日产5000吨干法水泥、株玻浮法三线等续建项目建设，促进南方公司和608所中小航空发动机基地、电力机车公司大功率重载交传机车等重大项目早日启动。发展壮大轨道交通装备制造、新材料等产业，进一步巩固提升株洲作为全省先进制造业中心的地位。同时，依托资源优势，提升烟花、陶瓷、纺织、农产品加工等产业。积极培育产业集群。坚持“产业必须集群发展，集群必须依托园区”的工作思路，认真落实各项优惠政策，继续拼盘投入4000万元支持园区建设和发展，增强园区的吸引力和承载力，放大园区的产业集聚和示范带动效应。立足产业定位，积极引导企业向专业园区和特色城镇集中，推动资源整合，延伸产业链条，加快培育专业化、特色化、规模化、集约化的产业集群。在支持建好原有18个园区的同时，全面推进欧洲工业园、清水塘循环经济工业区建设，努力把各个园区建设成为我市改革创新的“试验田”、对外开放的“窗口”和经济发展的重要增长极。加快繁荣现代服务业。优化城乡商业网点布局，全面推进商贸服务业现代化。支持启动炎陵神农中药文化纪念馆建设，整合旅游资源，提升炎帝历史人文旅游品牌，确保成功创建“中国优秀旅游城市”。支持发展现代物流等其他服务业。

　　改善基础设施，大力推进农业产业化。一方面，加快农业结构调整。稳定粮食生产，实施种苗工程，扩大特色种养，发展优质高效农业。壮大竹木、优质米、油脂、肉禽、果蔬等优势产业，规划建设规模农产品基地。扶持唐人神、太子奶等一批农产品加工企业，全年实现农产品加工产值65亿元。另一方面，改善农村基础设施。全面完成水利建设“34111”工程，支持洮水水库建设。退耕还林8.2万亩，森林覆盖率提高到57.9%。推进网朱公路建设，开工建设S320茶陵县界首至界化垅公路。继续做好S313醴陵至伞铺公路前期工作。加快乡村道路建设，建成农村客运站15个。继续推进农村电网改造。

　　完善配套功能，大力推进城镇化。城市快速环道全线贯通。加快湘江四桥、沿江防洪景观道路城区河西段、建宁港综合治理项目建设进度。启动建宁大道、新塘路、铁东路南段、湘江五桥和沿江防洪景观道路城区河东段建设，争取拉通泰山路。完成天鹅湖治理；新建和改造一批城市公厕和垃圾中转站。加快国家园林城市创建步伐。健全社区服务机制，提高社区服务水平。启动路灯微机控制系统建设，推行城市亮化工程智能化管理。增加公交车辆和营运线路，方便群众出行。新增享受经济适用房和廉租房补贴政策的家庭800户，住房公积金覆盖面扩大5%。深化户籍制度改革，合理引导农村人口向城镇集中。同时，支持株洲航电枢纽、武广客运专线和醴潭、长株、衡炎3条高速公路建设。

　　项目是后劲的依托、财税的源泉、产业的载体。无论是推进工业化、农业产业化，还是推进城镇化，无论是城市，还是农村，都必须牵住项目这个“牛鼻子”。一是在“争”字上下功夫，争取已纳入规划的项目尽早启动，争取更多的项目进入国家、省规划“笼子”。二是在“引”字上下功夫，认真策划、开发、推介项目，吸引更多的国外、市外战略投资者来我市投资置业，共谋发展。三是在“激”字上下功夫，激发民间投资活力，促进扩大社会投入，大力培育本土企业。四是在“融”字上下功夫，推动银企合作，拓宽金融支持领域，扩大信贷投放规模。全年力争完成全社会固定资产投资188亿元，实现两天投资1亿元，努力以大项目带动大投入，以大投入促进大发展。

　　（二）注重与时俱进，推进改革开放

　　深化各项改革。做好已启动市属国有企业改革的扫尾工作，继续支持在株中央、省属国有企业改革。巩固农村税费改革成果，全面推进农村综合改革，加快乡村消赤减债步伐。鼓励发展各类专业合作组织。加快城市公用事业市场化改革，探索特许经营路子。深化部门预算、国库集中支付、政府采购和收支两条线管理等改革，健全公共财政体系。推行相对集中行政处罚权，创新城市管理体制，提高城市管理水平。全面铺开事业单位管理体制改革。加快政企、政事、政资、政府与中介组织“四分开”步伐。进一步完善资本市场，活跃产权市场，健全技术、人才、劳动力市场。

　　扩大对外开放。创新招商引资方式，着力引进一批大项目、好项目，力争全年新批外资企业86家，实际利用外资1.8亿美元，引进内资70亿元。大力实施“出口名牌工程”，鼓励引进先进成套设备和先进技术，全年实现进出口总额7.5亿美元，增长13%，其中出口6.29亿美元，增长13%。支持企业“走出去”，进一步扩大对外经济技术合作，外派劳务500人以上。

　　壮大非公经济。全面确立非公企业平等的市场主体地位，努力形成“经济以民有为主，企业以民营为主，投资以民间为主，事业以民办为主”的发展格局。牢固树立“非禁即入”的准入观和一视同仁的支持观，鼓励参与国有企业改革、城乡基础设施建设和农业产业化经营，努力拓展非公经济的发展空间。对贡献突出的科技型、农产品加工型、外向型、就业安置型非公企业，切实加大财税支持力度。支持开展教育培训，引导非公企业诚信经营，规范发展，自觉维护职工合法权益。

　　（三）突出以人为本，推动社会进步

　　发展社会事业。完善科技成果转化激励机制，力争高新技术产业产值超过240亿元，增长20%。巩固基础教育，提升中职教育，发展高等教育，落实“两个比例、三个增长”，加大农村义务教育投入；促进民办教育快速发展；加大高技能人才培养力度，组建技师学院，支持铁道职院争创国家级示范性高职院校；积极推进湖南工业大学二期工程建设。加大医疗服务体系建设力度，改造35个乡镇卫生院，建好19个社区卫生服务中心和41个服务站。继续开展“周周乐”文化活动，整治网吧、图书、音像和软件版权市场，推进“绿色上网”。开展全民健身活动，提高竞技体育水平，举全市之力建好体育中心，承办好省十运会。

　　维护社会稳定。认真做好就业和社会保障工作，高度关注社会弱势群体。继续开展“平安株洲”创建活动，进一步改善社会治安环境。开展“五五”普法教育，增强市民法制意识。认真做好信访和人民调解工作。切实抓好安全生产，防止发生重特大事故。

　　做好人口资源环境工作。继续实施农村计生家庭奖励扶助政策，对农村计生困难家庭实行救助。保持耕地占补平衡，依法整顿和规范矿产资源开采秩序。切实加强植树造林、荒山治理和水土保持工作。编制《株洲市循环经济发展规划》，支持抓好株冶、智成化工两个国家级试点企业建设，申报实施一批循环经济项目，力争万元GDP能耗降低3%。继续开展“蓝天行动”，加大城区环境污染综合整治力度，市区工业企业污染物实现达标排放，全年空气良好天数在270天以上。

　　努力办好10件实事。1、社会保障体系建设。养老、失业、医疗保险参保人数分别新增5.47万人、2.45万人、3.2万人；新建和改造乡镇敬老院20所，集中供养五保户增加到3000人；城镇“低保”实现应保尽保，启动醴陵、茶陵农村“低保”试点；农村特困救助覆盖面继续保持100%。2、就业再就业工作。新增城镇就业4.7万人，下岗失业人员再就业2.3万人，“4050”人员再就业0.5万人，培训下岗失业人员1.5万人；农民职业技能培训1.2万人次，农村实用技术培训10万人次，农村劳动力转移就业新增5万人。3、教育事业发展。推进农村中小学寄宿制学校建设，资助9万名城乡中小学贫困生就学，市区新增“学位”4500个。4、食品安全检测体系建设。县（市）各建成1家超市食品安全自检室，全市建成50个市场运行监测点。5、社会主义新农村建设。组织编制乡村建设规划，每个县（市、区）建设一批新农村试点村。6、农村卫生服务体系建设。巩固醴陵新型农村合作医疗试点成果，启动炎陵、茶陵两县试点工作；全面实施农村大病医疗救助制度。7、农村水利建设。治理100座病险水库，解决1万名农村居民饮水困难。8、农村交通能源建设。完成“通达工程”100公里、“通畅工程”300公里，新建沼气池6000个。9、国家园林城市创建工作。新增城市绿地面积35.2万平方米，创建25家园林式单位（小区），提升25条道路绿化水平，绿地率、绿化覆盖率分别提高到35.4%、38.9%，人均公共绿地面积增加到8.3平方米。10、城区环境整治。改造10条城区支路和小街小巷，完善城市地下给排水管网系统，解决好合泰涵洞等地区的雨水排放不畅问题。

　　（四）转变政府职能，加强自身建设

　　优化发展环境。在改善“硬”环境的同时，着力治理发展“软”环境。市委、市政府将今年确定为“企业服务年”，务求政府为企业服务提高到一个新水平。重点把握这几个环节：深入企业开展帮扶活动，协助制定发展规划；拓宽企业融资渠道，协调开展银企合作，帮助企业申报项目、争取资金；精简审批事项，规范审批行为，严格办事时限制；加强政务服务中心建设，实行“一门受理，全程跟踪，一站式服务”；加大行政效能监察力度，充分发挥“经济89110”的作用，严肃查处各类破坏发展环境的行为。同时，大力打击偷税漏税、无照经营、欺行霸市等不法行为，整顿和规范市场经济秩序。深入开展金融安全区创建活动，推进社会信用体系建设，努力打造诚信株洲。

　　提高执政水平。树立正确的政绩观，更加重视社会管理、公共服务，切实解决人民群众关注的热点难点问题。创新行政管理方式，把政府该管的事一定管好，把政府不该管的事交还企业、市场和中介组织。加快政府系统电子政务建设，积极推进政务信息化进程。深入贯彻《行政许可法》，加强行政监察、财政监督、审计监督和政务督查，严肃查处和纠正违法违纪行为，建设一支廉洁、务实、高效的公务员队伍。

　　把握角色定位。全面实施市委的决策部署。认真执行市人大及其常委会的决议和决定，尊重人民政协、各民主党派、工商联以及其他社会各界人士的意见，加强与法院、检察院、军队等国家机关以及工会、共青团、妇联等群众团体的联系。真诚接受法律监督、民主监督和社会监督。

　　各位代表，同志们，面向“十一五”，我们站在了一个新的发展起点上。承前启后、继往开来，是我们庄严而神圣的使命。让我们在市委的坚强领导下，解放思想，与时俱进，开拓创新，励精图治，为创造株洲更加美好的未来而努力奋斗!
